# Ultrasound-guided Deep Iliacus Plane Block (DIPB): Cadaveric Evaluation and Pilot Retrospective Evaluation of Another Novel Fascial Plane Block for Hip Analgesia

**DOI:** 10.4274/TJAR.2025.252252

**Published:** 2026-02-09

**Authors:** Serkan Tulgar, Bahadır Çiftçi, Bediha Koyuncu, Ali Ahıskalıoğlu, Selçuk Alver, Bora Bilal, Bayram Ufuk Sakul, Ebru Otu, Madan Narayanan, Hacı Ahmet Alıcı

**Affiliations:** 1 Bahçeşehir University, Medical Park Göztepe Hospital, Department of Anaesthesiology and Reanimation, İstanbul, Türkiye; 2 İstanbul Medipol University Faculty of Medicine, Department of Anatomy, İstanbul, Türkiye; 3 İstanbul Medipol University Faculty of Medicine, Department of Anaesthesiology and Reanimation, İstanbul, Türkiye; 4 Atatürk University Faculty of Medicine, Department of Anaesthesiology and Reanimation, Development and Design Application and Research Center, Erzurum, Türkiye; 5 Biruni University Faculty of Medicine Hospital, Department of Anaesthesiology and Reanimation, İstanbul, Türkiye; 6 İstinye University Liv Hospital Topkapı, Department of Anaesthesiology and Reanimation, İstanbul, Türkiye; 7 Frimley Park Hospital NHS Foundation Trust, Consultant-Anaesthetics and Intensive Care, Camberley, United Kingdom; 8 İstanbul Medipol University Faculty of Medicine, Department of Pan Medicine, İstanbul, Türkiye

**Keywords:** Cadaveric study, deep iliacus plane block, hip analgesia, hip fracture, PENG block

## Abstract

**Objective:**

Regional anaesthesia for hip surgery aims to cover both articular and cutaneous nerves. Current techniques often miss components or require multiple injections. We hypothesized that the deep iliacus plane block (DIPB)—which involves injection deep to the iliacus muscle at the anterior inferior iliac spine—could simultaneously target both lumbar plexus branches and articular nerves.

**Methods:**

We conducted a cadaveric investigation and a retrospective clinical pilot. Bilateral DIPB was performed on a fresh cadaver (50 mL dye) using 50 mL of dye to assess dye spread. Clinically, 20 hip fracture patients received a single-injection DIPB (30-40 mL of 0.25% bupivacaine). Blocks were performed postoperatively (n = 13) or preoperatively for positioning (n = 7). Primary outcomes included dye spread and opioid consumption. Pain scores were evaluated before and after the block in the positioning subset.

**Results:**

Cadaveric dye stained the lateral femoral cutaneous nerve (LFCN), the femoral nerve (FN), and the pericapsular branches. In the clinical cohort (n = 20), the median postoperative numeric rating scale (NRS) score was 1; only one patient required rescue analgesia within 24 hours. In the positioning subset (n = 7), median NRS dropped from 9.0 (7-10) to 1.0 (0-2) 30 minutes post-block (*P* < 0.001).

**Conclusion:**

Preliminary findings suggest that DIPB may provide simultaneous coverage of the LFCN, FN, and pericapsular branches with a single injection. Further prospective studies are required to confirm the safety and efficacy.

**Main Points**

• This study introduces the deep iliacus plane block (DIPB), a novel single-injection technique hypothesized to provide simultaneous blockade of the pericapsular nerves that supply the hip capsule and of the major cutaneous nerves arising from the lumbar plexus.

• Anatomical evaluation in a cadaveric dissection demonstrated that the injectate stained the lateral femoral cutaneous nerve, the femoral nerve, and the articular branches supplying the hip capsule, thereby supporting the technique’s proposed mechanism.

• In the retrospective pilot study of 20 patients undergoing hip fracture repair, the DIPB showed a significant analgesic effect, reducing the median numeric rating scale pain score from 9.0 before the block to 1.0 thirty minutes after the block.

• The block provided sustained analgesia, with only one of the 20 patients requiring rescue analgesia within the first 24 hours postoperatively.

## Introduction

Hip surgeries require effective regional anaesthesia to optimize perioperative analgesia and facilitate patient positioning. Over the past few decades, clinical research has increasingly focused on refining these techniques.^1^ The lumbar plexus plays a crucial role in providing anaesthesia and analgesia for hip and knee procedures. It consists of the obturator nerve, lateral femoral cutaneous nerve, and femoral nerve. Together with the sacral plexus, it provides innervation to the lower limb.^2^

The suprainguinal fascia iliaca block (SIFIB) is a regional technique designed to block the anterior components of the lumbar plexus.^3^ However, it requires a relatively high volume of local anaesthetic,^4^ which increases the risk of direct quadriceps weakness. Recently, the Pericapsular Nerve Group (PENG) block has emerged to selectively block the articular branches of the femoral, obturator, and accessory obturator nerves supplying the hip capsule.^5^ While the PENG block may provide superior analgesia compared to a femoral nerve block alone,^6^ high volumes (>30 mL) can lead to motor weakness due to spread into the psoas compartment.^7, 8^ Another proposed technique is the iliopsoas plane block (IPPB); however, this approach requires the identification of a deep fascial plane that can be technically demanding.^9^

Current literature indicates that while the SIFIB provides a broad cutaneous blockade, it may spare the deep articular branches and the obturator nerve.^1, 4^ Conversely, PENG and IPPB target these deep branches but often fail to block the lateral femoral cutaneous nerve and the femoral nerve, which provide cutaneous innervation to the incision site.

We hypothesized that by injecting a sufficient volume of local anaesthetic deep to the iliacus muscle at the level of the anterior inferior iliac spine, we could achieve simultaneous blockade of both the cutaneous branches of the lumbar plexus and the PENG. We aimed to present this novel technique, deep iliacus plane block (DIPB), via a cadaveric evaluation of injectate spread and a pilot retrospective study assessing its potential analgesic effect.

## Methods

The İstanbul Medipol University Ethics and Research Committee approved (approval no.: 965, date: 31.07.2025) the retrospective evaluation of 20 patients who underwent DIPB. The cadaveric examination was conducted with the approval of the same Institutional Review Board (IRB) (approval no.: 65, date: 18.01.2024). The IRB waived the requirement for written informed consent.

One fresh-frozen cadaver specimen (female, 63 years of age) was included. The specimen demonstrated normal anatomy and showed no evidence of previous surgical procedures, trauma, or pathological changes involving the inguinal or lower abdominal regions.

### Description of DIPB

**Figure 1:**
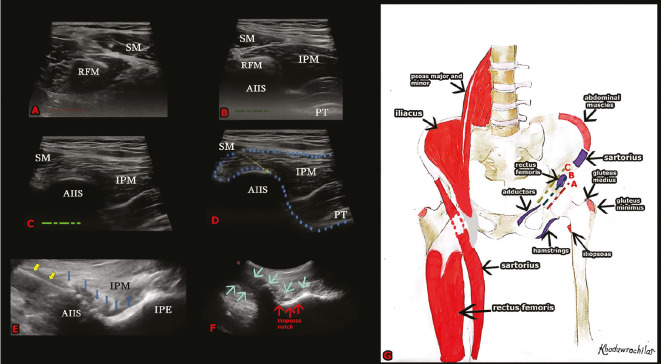
Sonographic anatomy and anatomic illustration of deep iliacus plane block. A: Sartorius and rectus femoris muscles are seen in the caudal direction. B: As the probe moves cranially, the sartorius, rectus femoris, iliopsoas muscle, iliopsoas tendon, and anterior inferior iliac spine are visualized. C: Rectus femoris is out of view. Sartorius and iliopsoas muscles, iliopsoas tendon, and AIIS are seen D: The yellow arrow indicates the direction of the needle toward the AIIS. The area enclosed by blue dots denotes the block’s target area. E: The Spread of local anaesthetic is evident. The yellow arrows indicate the needle. The blue arrows indicate the spread of lo-cal anaesthetic. F: The spread of local anaesthetic is seen along the iliopsoas notch. The blue arrows indicate the spread of local anaes-thetic.G: Anatomic illustration of the muscles and attachment points in the block area. The sartorius muscle is cut to reveal the attachment point of the rectus femoris muscle. SM, sartorius muscle; RFM, rectus femoris muscle; IPM, iliopsoas muscle; AIIS, anterior inferior iliac spine; PT, psoas tendon.

With the cadaver in the supine position, blocks were performed. The convex transducer (Clarius, Canada), in trapezoid imaging mode, was positioned obliquely from superolateral to inferomedial, just above the femoral crest, similar to the PENG block technique. Anatomical structures, including the AIIS, iliopubic eminence (IPE), sartorius muscle (SM), iliopsoas muscle (IPM), psoas tendon, femoral nerve, femoral artery, femoral vein, and iliac fascia, were identified sono-anatomically. The anterior inferior iliac spine was centered in the transducer image. To determine the insertion point of the rectus femoris tendon (RFT) on the anterior inferior iliac spine, the transducer was rotated sagittally, and the level where the RFT ends cephalically was identified. After identifying the target (the most cephalic level between anterior inferior iliac spine and IPM without tendon-bursa), the transducer was obliquated again to visualize the IPE. The 22G x 100 mm block needle (Stimuplex Ultra 360, B-Braun, USA) was advanced in-plane from lateral to inferomedial towards the potential space between the IPM and the anterior inferior iliac spine. After confirming the target plane with a few mL of saline, 50 mL of 0.5% methylene blue solution was applied to the area (Figure 1). We used 50 mL of dye in the cadaveric model to clearly delineate the maximum anatomical spread of the injectate, a standard volume for cadaveric dye studies.

Unfortunately, although identification of the insertion point and tendon of the rectus femoris muscle (RFM) on the left side was successful, the insertion of the RFM and its tendon could not be identified on the right side; therefore, the anterior inferior iliac spine was targeted directly. The spread could not be clearly determined.

### Cadaveric Dissection

Approximately one hour after block performance, two experienced anatomists initiated bilateral dissections. The dissection line was drawn from the anterior inferior iliac spine to the tuberculum pubicum, and from the midpoint of that line to the midpoint of the patella. Dissection of the skin and fascia started at the midline and proceeded laterally. The SM and lateral femoral cutaneous nerve were examined for staining. After the femoral nerve was identified in the femoral triangle, the muscular branches of the femoral nerve and the subsartorial canal were exposed by dissecting the SM from its insertion. Afterward, the tendon of the IPM was dissected from the insertion point, the hip joint capsule was exposed, and the presence of staining in the articular branches of the obturator and femoral nerves was evaluated.

### Pilot Study Assessing the Potential Analgesic Effect of DIPB

This cadaver study was performed on a single model; subsequently, the authors obtained informed consent from willing patients for this block. Patients who underwent DIPB between February 2024 and August 2025 were evaluated for opioid consumption and pain scores. Written informed consent, which specifically included consent for administration of the novel interfascial plane block technique, was obtained from all patients. All operations were performed by the same surgical team. All blocks were performed by the same anaesthesiologists (the authors) to minimize technical variability. Pain assessments were performed by a nurse anaesthetist not affiliated with the study team. For spinal anaesthesia, a standardized dose of 10-12.5 mg of hyperbaric bupivacaine was administered, in accordance with our institution’s protocol for hip fracture surgery. As this was a retrospective analysis of clinical cases, the injectate volume was not fixed but ranged from 30 to 40 mL. This volume was determined by the attending clinician, based on the patient’s body weight, to ensure safety with respect to local anaesthetic systemic toxicity.

**Figure 2:**
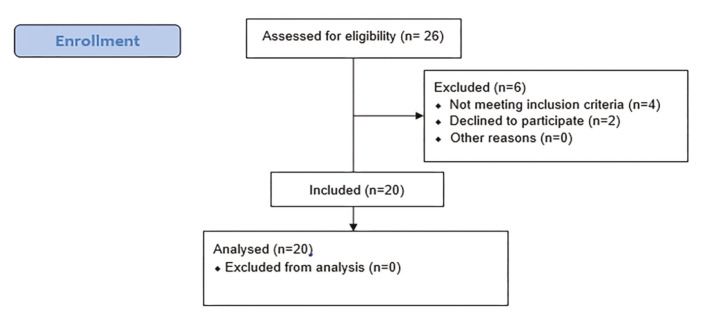
CONSORT flow diagram of the retrospective evaluation.

Based on the cadaveric findings, DIPB was chosen for eligible patients who were likely to benefit from this novel technique. Patients were included in this retrospective analysis if they were adults (≥18 years old) undergoing hip fracture repair surgery, were classified as American Society of Anesthesiologists (ASA) physical status II or III, and had received a single-injection DIPB for postoperative analgesia. Patients were excluded if they had pre-existing neurological deficits affecting the lower limbs, a known allergy to local anaesthetics, or severe coagulopathy. Figure 2 shows the CONSORT flow chart used for patient enrollment.

The block was performed in the supine position under sterile conditions. All DIPBs were performed using a low-frequency convex transducer with an in-plane approach. After sterile preparation and local infiltration of the skin, the needle tip was advanced under real-time ultrasound guidance to the fascial plane deep to the iliacus muscle at the level of the anterior inferior iliac spine. After confirmation of the proper block area with 5 mL saline, 30-40 mL of 0.25% bupivacaine was administered. The Spread of local anaesthetic was visualized within the target plane. The clinical technique mirrored the cadaveric approach. However, for patient safety, the attending anaesthesiologist reduced the volume to 30-40 mL of 0.25% bupivacaine based on each patient’s weight and safety profile.

Eight patients underwent general anaesthesia, and 12 underwent spinal anaesthesia. Patients receiving general anaesthesia received the standard general anaesthesia protocol. After monitoring (ECG, arterial pressure, SpO₂), induction was performed with IV propofol (1-2 mg kg^-1^), fentanyl (2-5 µg kg^-1^), and followed by rocuronium (0.6-0.8 mg kg^-1^) for intubation. Spinal anaesthesia was administered under sterile conditions with the patient in the lateral decubitus position with the fractured side on top. A 25 G spinal needle was inserted into the L3-L4 or L4-L5 interspinous space and 10-12.5 mg of bupivacaine heavy was injected. The surgery was started after successful spinal anaesthesia is con-firmed by a dermatome test.

All of the blocks were unilateral. We performed DIPB in seven patients for positional pain before spinal anaesthesia. We recorded the pain scores before and 30 min after the block, and during positionbefore the block, during positioning, and 30 min after the block. We ordered 400 mg of intravenous ibuprofen for the patients every 8 hours during the postoperative period. We planned to administer 100 mg of tramadol as a rescue analgesic if the patient’s NRS score was above 4. We observed all patients for 24 hours postoperatively.

### Statistical Analysis

Statistical analyses were performed using IBM SPSS for Windows, version 20.0 (SPSS, Chicago, IL, USA). To evaluate the assumption of normality, the Shapiro-Wilk test was employed. Numerical variables were presented as mean ± standard deviation or as median (25^th^-75^th^ percentiles), depending on data normality. A t-test and a signed-rank test were performed to evaluate the differences between the measurements. *P *< 0.05 was considered statistically significant.

## Results

### Cadaveric Findings

On the right side, the injectate stained only the RFM. No dye was detected outside the fascia surrounding the RFM.

**Figure 3:**
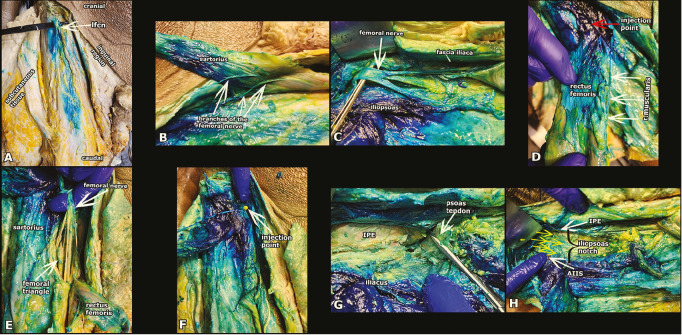
Dye spread of the deep iliacus plane block. A: The staining of the lateral femoral cutaneous nerve was observed. The white arrow indicates the LFCN. B: The sartorius muscle was removed. Just below the sartorius, stained branches of the femoral nerve are visible. The white arrows indicate branches of the femoral nerve. C: Below the fascia iliaca, the stained iliopsoas muscle and the posterior and anterior divisions of the femoral nerve are seen. The white arrow indicates the femoral nerve. D: The rectus femoris muscle is manually lifted. Dye spread is seen in the nervus rectus femoris and its branches, deep to the rectus femoris muscle in the femoral triangle. The white arrows indicate the r. muscularis of the femoral nerve. The red arrow indicates the injection point of the block. E: The femoral nerve is seen in the femoral triangle. There is intense staining on the femoral nerve and adjacent anatomical structures. F: This image demonstrates intense staining in the block area. The white arrow indicates the injec-tion point. E: Extensive dye spread is seen between the IPE and the iliacus muscle, deep to the psoas tendon. This region corresponds to the area where the iliopsoas plane block is performed. The white arrow indicates the psoas tendon. F: Dye spread was observed in the area corresponding to the iliopsoas notch between the IPE and AIIS. Yellow arrows indicate the articular branches of the femoral nerve. LFCN, lateral femoral cutaneous nerve; IPE, iliopubic eminence; AIIS, anterior inferior iliac spine.

Dissection of the skin and subcutaneous tissue on the left revealed conspicuous staining of the lateral femoral cutaneous nerve adjacent to the SM. The deep fascia of the SM and the branches of the femoral nerve supplying this area were also stained. The spread of injectate was observed beneath the IPM, below the psoas tendon, and around the iliacus muscle within the iliac fascia. Upon lifting the SM and the fascia iliaca, the surface of the IPM and both the posterior and anterior divisions of the femoral nerve were stained. This staining was prominent in the femoral nerve, which runs deep to the RFM and in its branches within the femoral triangle. Extensive staining was also noted between the IPE and the iliacus muscle, deep to the psoas tendon, and caudally in the area where the IPPB was applied. The articular branches of the femoral nerve were observed to be stained within the iliopsoas notch. Cadaveric images and areas of intense methylene blue staining are presented in Figure 3.

### Clinical Patient Results

While all 20 patients were monitored for 24-hour postoperative opioid consumption, a subset of seven patients received the DIPB preoperatively to manage severe pain during positioning for spinal anaesthesia. For these seven patients, NRS pain scores were recorded immediately before the block and 30 minutes post-block. Among the 20 patients, 8 received general anaesthesia and 12 received spinal anaesthesia. We observed no discernible difference in postoperative pain scores or rescue analgesia requirements between these two subgroups.

Patient demographic and block characteristics are presented in Table 1. The study included 20 patients with a median age of 71.0 years (64.5-77.0), a median height of 161.5 cm (158.2-172.2), and a median weight of 73.5 kg (67.8-78.5). Of these patients, nine were male and eleven were female; ASA classifications were II (n = 14) and III (n = 6). The mean duration of surgery was 113.5 minutes (107.2-118.0). The mean duration of anaesthesia was 133.0 minutes (128.8-137.5). Eight patients underwent surgery under general anaesthesia, while 12 received spinal anaesthesia. Surgical procedures included total hip prosthesis in 13 patients (65.0%) and partial hip replacement in 7 patients (35.0%). The post-NRS scores are shown in Table 2.

Only one patient required rescue analgesia, specifically, 100 mg tramadol, at the 16^th^ postoperative hour. The remaining 19 patients did not require any additional analgesics within 24 hours post-block. No adverse effects, such as local anaesthetic systemic toxicity, nerve injury, or hematoma, were observed in any of the patients included in this pilot case series. The type of anaesthesia (general vs. spinal) did not appear to be a primary determinant of rescue analgesic needs, as patients in both anaesthesia groups experienced significant pain reduction and minimal need for rescue analgesics.

In seven patients, the median NRS score before the block was 9 (7-10), and the median NRS score 30 minutes after the block was 1 (0-2) (Table 3). A paired t-test showed a statistically significant difference between the two measurements (*P* < 0.001). No adverse effects, such as local anaesthetic systemic toxicity, nerve injury, or hematoma, were observed in any patient. Furthermore, no block failures were observed among the included patients, as evidenced by a significant reduction in pain scores after block placement.

## Discussion

We identified that “DIPB”, despite the absence of an anatomical plane in that region, suggests simultaneous coverage of the lateral femoral cutaneous nerve, the femoral nerve, and the PENG with a single injection. We were unable to advance to the obturator canal to visualize the obturator nerve, and no dye was observed beneath the pectineus muscle.

Regional anaesthesia techniques are frequently used in lower-extremity surgeries for both analgesia and anaesthesia. However, the only method to achieve complete blockade of all components of the lumbar plexus is through a lumbar plexus block performed in either the lateral or prone position.^2^ Positioning, particularly in trauma patients, can be challenging. Additionally, clinicians may avoid this deep and relatively complex technique in patients on anticoagulants or those with coagulation disorders.

Anterior techniques have limitations, and no technique guarantees blockade of both the articular and cutaneous branches of the nerves of the lumbar plexus. For this purpose, a combination of PENG block and the lateral femoral cutaneous nerve block can be used10, or a high-volume SIFIB (60 mL) may be employed.^3, 11^ A study hypothesized that combining SIFIB and PENG blocks could achieve extensive spread. Cadaveric evaluations demonstrated that this combination could block major components and terminal branches of the lumbar plexus, except for the obturator nerve.^12^ In our cadaveric evaluation, we determined that we could achieve such widespread distribution, with the same volume, using a single injection.

The SIFIB successfully targets the articular branches of the femoral, obturator, and accessory obturator nerves that innervate the anterolateral hip joint and are often missed by the infra-inguinal approach because of their cephalad separation.^1^ However, achieving adequate blockade of the obturator nerve’s articular branch within the hip capsule may necessitate significantly higher volumes of local anaesthetic, which could cause direct weakness of the quadriceps muscle. The PENG block is not effective at providing cutaneous innervation of the surgical incision line in hip surgery. It has been argued that when the injected volume in the PENG block exceeds 30 mL, it may result in anteromedial spread to the psoas, producing effects similar to a fascia iliaca block and potentially causing motor weakness.^7, 8^ We are aware that the SIFIB, while providing a broad cutaneous blockade of the hip, it may miss deep branches to the hip capsule and the obturator nerve. PENG and IPPB target deep branches, but fail to block the lateral femoral cutaneous nerve and the femoral nerve, which are involved in cutaneous innervation.

The DIPB aims to address a specific clinical gap: the need for comprehensive hip analgesia via a single injection. Currently, the PENG block effectively targets the deep articular branches but often spares the superficial cutaneous nerves (LFCN and FN), potentially resulting in pain at the incision site. Conversely, the SIFIB provides excellent cutaneous coverage, but may not consistently reach the deep and accessory obturator articular branches. The DIPB is anatomically positioned to function as a hybrid, utilizing a high-volume injection deposited deep to the iliacus muscle to spread cephalad (targeting lumbar plexus roots, similar to SIFIB) and caudally (targeting articular branches, similar to PENG).

### Study Limitations

This study has several significant limitations. First, the anatomical evaluation was limited to a single cadaver. Notably, the block failed on the cadaver’s right side due to poor visualization of the rectus femoris tendon, highlighting a learning curve and the risk of sono-anatomical misinterpretation. Second, our clinical data are retrospective, have a small sample size (n = 20), and lack a control group. Third, we did not systematically evaluate quadriceps motor strength. The use of 30-40 mL of local anaesthetic carries a risk of motor blockade (quadriceps weakness) due to femoral nerve involvement, which we observed during dissection. This necessitates caution in patients requiring early ambulation. Finally, variation in anaesthesia type (spinal vs. general) may have influenced perception of postoperative pain.

## Conclusion

The DIPB is a novel technique that may offer simultaneous blockade of the lumbar plexus cutaneous branches and hip articular nerves. While our pilot data indicate effective analgesia for hip fracture patients, the risk of motor block and the technical difficulty of the injection require further investigation. Randomized controlled trials are needed to validate these preliminary findings.

## Ethics

**Ethics Committee Approval:** The İstanbul Medipol University Ethics and Research Committee approved (approval no.: 965, date: 31.07.2025) the retrospective evaluation of 20 patients who underwent DIPB. The cadaveric examination was conducted with the approval of the same Institutional Review Board (approval no.: 65, date: 18.01.2024).

**Informed Consent:** Retrospective study.

## Figures and Tables

**Table 1. Demographic Data of the Patients:** 

**Gender (M/F)**	9/11
**Age**	71.0 (64.5-77.0)
**Height (cm)**	161.5 (158.2-172.2)
**Weight (kg)**	73.5 (67.8-78.5)
**ASA I/II/III**	0/14/6
**Duration of surgery (min)**	113.5 (107.2-118.0)
**Duration of anaesthesia (min)**	133.0 (128.8-137.5)
**Operation type**	Total hip prosthesis: 13 (65.0%); partial hip replacement: 7 (35.0%)
**Incision type**	Posterolateral incision: 14 (70.0%); lateral incision: 5 (25.0%); anterior incision: 1 (5.0%)
**Anaesthesia type**	Spinal anaesthesia: 12 (60.0%); general anaesthesia: 8 (40.0%)

Values are expressed as median (percentiles 25-75) or number

ASA, American Society of Anesthesiologist; cm, centimeter; F, female; kg, kilogram; M, male; min, minutes

**Table 2. NRS Scores at 1, 4, 8, 16, and 24 h Postoperatively:** 

**Hours**	**Median values (min-max)**	**Mean**
1^st^ hour	0 (0-2)	0.45
4^th^ hour	0 (1-2)	0.7
8^th^ hour	0 (1-2)	1.2
16^th^ hour	0 (1-3)	1.15
24^th^ hour	0 (1-1)	0.7
1^st^ hour	1 (1-3)	1.4
4^th^ hour	1 (2-3)	1.8
8^th^ hour	1 (2-3)	2.1
16^th^ hour	1 (1-5)	1.8
24^th^ hour	0 (1-2)	1.2

Data are expressed as median (percentiles 25-75)

NRS, numeric rating pain scale; min-max, minimum-maximum

**Table 3. Comparison of the Before Block and After Block NRS Scores and Evaluation of NRS Values During Position:** 

-	**Median values (min-max)**	**Mean**
NRS before block (n = 7)	9 (7-10)	8.7
NRS after block (n = 7)	1 (0-2)	1
NRS during position (n = 7)	2 (1-3)	2.2

*P* value for the comparison of the scores between “before” and “after” block =0.0000034

*P* value is obtained with paired t-test (n)

NRS, numeric rating scale; min-max, minimum-maximum
